# The role of Eya1 and Eya2 in the taste system of mice from embryonic stage to adulthood

**DOI:** 10.3389/fcell.2023.1126968

**Published:** 2023-04-25

**Authors:** Ting Zhang, Pin-Xian Xu

**Affiliations:** ^1^ Department of Genetics and Genomic Sciences, Icahn School of Medicine at Mount Sinai, New York, NY, United States; ^2^ Department of Cell Developmental and Regenerative Biology, Icahn School of Medicine at Mount Sinai, New York, NY, United States

**Keywords:** Eya1, Eya2, tongue, taste bud, type II taste cells, circumvallate and foliate papillae

## Abstract

Members of the Eya family, which are a class of transcription factors with phosphatase activity, are widely expressed in cranial sensory organs during development. However, it is unclear whether these genes are expressed in the taste system during development and whether they play any role in specifying taste cell fate. In this study, we report that *Eya1* is not expressed during embryonic tongue development but that *Eya1*-expressing progenitors in somites or pharyngeal endoderm give rise to tongue musculature or taste organs, respectively. In the *Eya1*-deficient tongues, these progenitors do not proliferate properly, resulting in a smaller tongue at birth, impaired growth of taste papillae, and disrupted expression of *Six1* in the papillary epithelium. On the other hand, *Eya2* is specifically expressed in endoderm-derived circumvallate and foliate papillae located on the posterior tongue during development. In adult tongues, *Eya1* is predominantly expressed in IP_3_R3-positive taste cells in the taste buds of the circumvallate and foliate papillae, while *Eya2* is persistently expressed in these papillae at higher levels in some epithelial progenitors and at lower levels in some taste cells. We found that conditional knockout of *Eya1* in the third week or *Eya*2 knockout reduced Pou2f3^+^, Six1^+^ and IP_3_R3^+^ taste cells. Our data define for the first time the expression patterns of *Eya1* and *Eya2* during the development and maintenance of the mouse taste system and suggest that *Eya1* and *Eya2* may act together to promote lineage commitment of taste cell subtypes.

## Introduction

The tongue, a muscular organ that manipulates food for chewing and swallowing, is one of the primary organs of taste. The tongue’s upper surface is covered with sensory taste buds, which are housed in the papillae. In mammals, there are three different types of taste papillae: fungiform papillae (FUP), which are located over the anterior two-thirds of the tongue, and circumvallate (CVP) and foliate (FOP) papillae, which are situated at the back of the tongue (Witt, 2019). While a single CVP is located in the midline of the tongue, the FOP consists of inwardly folded epithelial trenches on both sides. Each taste bud forms an onion-like shape and is composed of distinct types of cells, such as taste receptors (TRCs) that span the depth of the papilla epithelium and supporting cells (Finger and Barlow, 2021). The TRCs are maintained by a continuously renewing population of progenitor cells that reside along the basement membrane of the epithelium (Barlow and Klein, 2015). The taste buds that receive gustatory stimuli are innervated by cranial nerves that respond to chemicals from food transmitted through taste receptor cells, resulting in the sensations of salty, sour, bitter, sweet, and umami (Northcutt, 2004).

While taste bud differentiation is induced around or after birth, taste papilla development in mice begins around embryonic day (E) 12.5, when taste primordia emerge as epithelial thickenings or taste placodes, which undergo dynamic morphogenetic events of evaginations and invaginations to form the epithelial papillae. There are 70–90 FUPs on the anterior tongue, and each contains a single taste bud, which differentiates as clusters of TRCs in the center of the papillary epithelium around birth (Kim et al., 2003; Mistretta and Liu, 2006). In contrast, taste buds in the CVP and FOP develop postnatally. They originate initially in the papillary region and later in the trench epithelium, formed by the invagination of the epithelium into the underlying mesenchyme beginning around E14.5. These trench epithelial cells undergo an extensive growth period to generate sufficient progenitor cells before the induction of taste cell differentiation. From the base of trenches, minor salivary glands (von Ebner’s glands) develop to aid gustatory sensation (Lee et al., 2006). However, how tongue epithelial progenitors are induced to diversify and generate different cell types in the taste system remains poorly understood.

Cell fate diversification is known to be controlled by gene expression programs that are regulated spatiotemporally by tissue- and cell type-specific transcription factors. Several transcription factors have been identified to play important roles in controlling the differentiation of embryonic tongue epithelium into taste papillae and the generation of different types of TRCs. Among them, Sox2 is an essential regulator of taste bud development and TRC generation and maintenance in adults (Okubo et al., 2006; Nakayama et al., 2015; Castillo-Azofeifa et al., 2018; Ohmoto et al., 2020). In contrast, Pou2f3 and Mash1 are critical for the generation of distinct TRC subtypes (Matsumoto et al., 2011; Seta et al., 2011). Previous studies have also suggested a role for the homeodomain Six/So (Sine oculis) protein family members Six1 and Six4 in the taste papillae during mouse embryonic development (Suzuki et al., 2010a; Suzuki et al., 2010b; Suzuki et al., 2011). However, it is not well understood how these transcription factors interact to spatiotemporally regulate the gene expression programs that establish and maintain distinct taste structures.

The Eya (eyes absent) protein family represents a class of transcription factors without DNA binding ability but with phosphatase activity. It encodes a divergent N-terminus possessing a transcriptional activation function (Xu et al., 1997a) and a highly conserved C-terminal Eya domain (ED) (Xu et al., 1997b) that participates in protein-protein interactions (Buller et al., 2001; Li et al., 2003; Rayapureddi et al., 2003; Tootle et al., 2003). We previously demonstrated that Eya1 interacts with chromatin remodelers and DNA-binding proteins Six1 and Sox2 to regulate cranial sensory organ development (Ahmed et al., 2012a; Ahmed et al., 2012b; Wong et al., 2013; Xu et al., 2021) or with Six1/Six2/Six4 to regulate kidney formation (Xu et al., 2003; Xu, 2013; Xu et al., 2014; Xu and Xu, 2015; Li et al., 2021; Li et al., 2022; Xu et al., 2022). Deletion of either *Eya1* or *Six1* in mice results in similar organ defects (Xu et al., 1999; Zheng et al., 2003; Ozaki et al., 2004; Zou et al., 2004; Zou et al., 2006a; Zou et al., 2008; Ahmed et al., 2012a; Ahmed et al., 2012b; Xu et al., 2021), while mutations in either gene in humans cause branchio-oto-renal syndrome (Abdelhak et al., 1997a; Abdelhak et al., 1997b; Ruf et al., 2004; Shah et al., 2020). *Eya2*, another member of the Eya family, is also widely expressed in sensory structures during development, including the sensory nervous system, olfactory epithelium, inner ear sensory organs, and salivary glands, in a complementary or overlapping pattern with that of Eya1 (Xu et al., 1997b; Ishihara et al., 2008; Chen et al., 2009; Zhang et al., 2021). *Eya2* knockout mice exhibit a normal appearance but have hearing loss (Zhang et al., 2021). In the taste system, RNA-seq analysis of chick oral tissues detected *Eya2* transcripts (Cui et al., 2017). However, a recent *in situ* hybridization study reported the expression of *Eya1* in subsets of taste bud cells in the CVP and FUP of mice aged 8–12 weeks, but not in FOP (Ohmoto et al., 2021). However, the expression patterns of these genes during the embryonic development of taste organs have not been studied. Furthermore, whether these genes play a role in taste organogenesis or TRC differentiation is not known.

In this study, we investigated the potential roles of *Eya1* and *Eya2* in the development and maintenance of the mouse taste system from the embryonic stage to adulthood. Specifically, we examined the expression of *Eya1* ^*LacZ*^ and *Eya2* ^*LacZ*^ knockin reporter alleles in the tongues by staining for β-Gal activity and investigated whether deletion of either gene disrupted taste organogenesis and taste cell generation. We found that although *Eya1* is not expressed in the tongue during embryonic development, *Eya1*-expressing progenitors in the pharyngeal endoderm or somites at E9.5 give rise to the lingual epithelium and epithelium-derived taste organs or tongue musculature, respectively. In *Eya1*-deficient tongues, these progenitor cells fail to proliferate normally, resulting in a smaller tongue at birth, impaired growth of taste papillae, and disrupted expression of Six1 in the papilla epithelium. After birth, *Eya1* expression is detectable from 1 week to adulthood. It is expressed in some basal progenitors and a subset of IP_3_R3^+^ TRCs of the endoderm-derived CVP and FOP in the posterior tongue but not in the ectoderm-derived FUP in the anterior tongue. *Eya1* conditional knockout (cKO) during the third week using *Sox2* ^*CreER*^ resulted in a reduced number of Type II TRCs. In contrast, we found that *Eya2* is expressed in the CVP and FOP from E14.5 shortly after the onset of their morphogenesis. Soon after birth, *Eya2* is expressed throughout the CVP and FOP trench epithelium. However, in the adult tongue, *Eya2* ^*LacZ*^ is expressed in some cells near the taste pore surrounding the taste bud and some basal precursors and taste cells, and the number of Type II TRCs is also reduced in *Eya2*-deficient mice. Together, our results define the expression patterns of *Eya1* and *Eya2* during the development and maintenance of the mouse taste system for the first time. Our results suggest that *Eya1* and *Eya2* may function together to promote the lineage specification of taste cell subsets in adult taste buds at the posterior tongue.

## Materials and methods

### Animals and genotyping

Mice carrying *Eya1* ^*+/−*^ (Xu et al., 1999), *Eya1* ^*LacZ*^ (Zou et al., 2008), *Eya1* ^*CreER*^ (Xu et al., 2014)*, Eya1* ^*Flox10/Flox10*^ *, R26* ^*LacZ*^ *, Eya2* ^*+/−*^ and *Eya2* ^*LacZ*^ (Zhang et al., 2021), and *Sox2* ^*CreER*^ (Arnold et al., 2011) were housed at the Icahn School of Medicine at Mount Sinai Animal Facility, on a mixed background of 129/Sv and C57BL/6J strains. *Eya1* ^*Flox10*^ was generated by Biocytogen through the use of LoxP sites to flank two exons—exons 10 and 11.

PCR genotype was performed as previously reported. All animal protocols were approved by Animal Care and Use Committee of the Icahn School of Medicine at Mount Sinai (protocol #06-0807).

### X-gal staining

Mice were anesthetized with isoflurane and transcardially perfused with phosphate-buffered saline (PBS), followed by 4% paraformaldehyde (PFA) in PBS. Tissue organs were then fixed with 4% PFA for 30 min, cryoprotected in 30% sucrose overnight, embedded in OCT compound (Tissue-Tek; Sakura Finetek USA, Torrance, CA), and sectioned on a cryotome at 6 μm thickness. Sections were air-dried, rinsed in PBS, and stained in a solution containing 0.8 mg/mL X-Gal in 35 mM K_3_Fe(CN)_6_, 35 mM MK_4_Fe(CN)_6_, 2 mM MgCl_2_ in PBS at room temperature until the blue staining appeared. Sections were then dehydrated through a series of graded ethanol, cleared in xylenes, and cover-slipped with Permount. For whole-mount staining, the embryos were fixed with 4% PFA for 30 min, rinsed in PBS, and stained with the same chemicals listed above.

### Histology and immunohistochemistry (IHC)

Histology and immunostaining were performed following standard procedures. Briefly, tongues were collected in ice-cold PBS and fixed in 4% PFA for 30 min at room temperature. Samples were cryoprotected in 30% sucrose at 4°C overnight and then submerged in OCT compound and flash frozen on dry ice. Frontal sections were generated at 6 μm. Some sections were pretreated with citrate buffer (10 mM, pH6.0) at 65°C for 3 h for antigen retrieval before IHC.

Primary antibodies used were before IHC rabbit anti-Sox2 (PA1-094, ThermoFisher), rabbit anti-Six1 (50-204-6211, Cell Signaling), mouse anti-TUBB3 (66375-1-Ig, Proteintech), rabbit anti-β-Gal (08559761, MP Biomedicals), rabbit anti-Pou2f3 (PA5-40556, ThermoFisher), mouse anti-IP_3_R3 (610312, BD Biosciences), rabbit anti-Ncam (ab220360, Abcam), and mouse monoclonal Keratin 8 (Krt8) (NBP2-44940, Novusbio). Cy3-or Cy2-conjugated secondary antibodies were used. Nuclear staining was performed using Hoechst 33342.

### Measurement of CVP taste crypt height

The height of taste crypts in serial sections across the entire papilla was measured for three CVPs of wild-type control and *Eya1* ^*−/−*^ littermates, respectively, using Photoshop software, and the measurements were plotted using Prism software.

### EdU and TUNEL assays and statistical analysis

The EdU labeling assay was performed using a kit (cat# C10640, Life Technologies) following the manufacturer’s instructions. EdU was injected 90 min before harvest. Serial sections from three wild-type (35 sections, 6 μm/section) and three *Eya1*-deficient (33 sections, 6 μm/section) E15.5 CVPs and FOPs were prepared, and EdU^+^ cells were counted in the epithelial trenches. Statistical significance was assessed using a two-tailed *t*-test.

The TUNEL assay was performed using the ApopTag kit for *in situ* apoptosis fluorescein detection (cat# NC9815837, Millipore) following the manufacturer’s instructions. TUNEL^+^ cells were counted in the CVP epithelium. Statistical significance was assessed using a two-tailed *t*-test.

## Results

### *Eya1*-null mice display smaller tongues at birth and abnormal development of taste papillae

**FIGURE 1:**
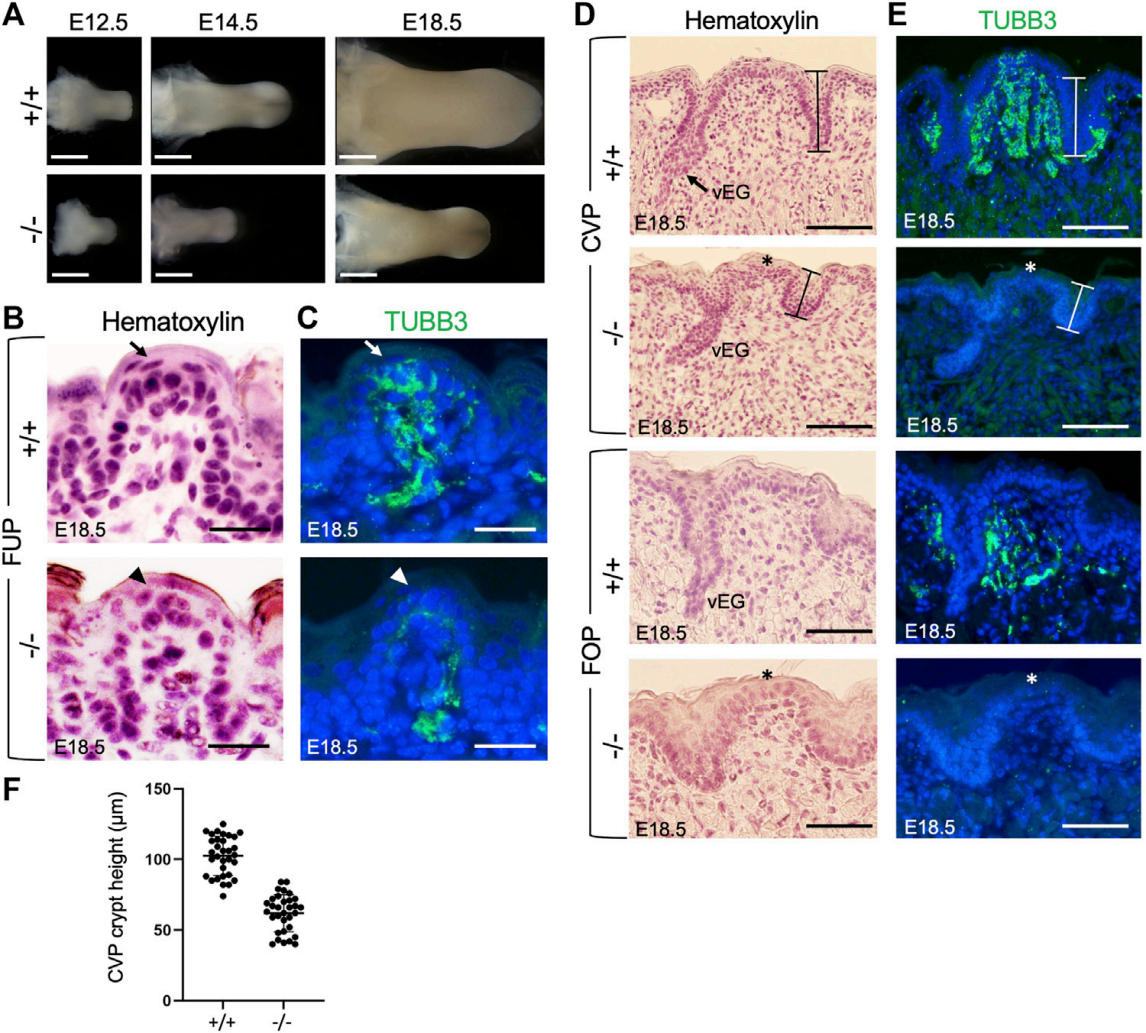
*Eya1* deficiency leads to growth arrest of tongue and taste papillae during development. **(A)** Tongues of wild-type and *Eya1*-null mice at E12.5, E14.5 and E18.5. **(B, D)** Hematoxylin-stained sections of E18.5 tongues showing FUP **(B)** and trenches of CVP and FOP with von Ebner’s glands (vEG) **(D)** in the wild-type and *Eya1* ^*−/−*^. **(C, E)** Immunostaining for anti-TUBB3 showing association of cranial gustatory nerves with papillae and trenches at E18.5, but reduced or lack of nerve contact in the mutant. Sections for FOP in D,E were sagittal, and all others were frontal. Arrows indicate well-developed taste buds with tightly clustered cells in wild-type FUP, and arrowheads indicate abnormal taste bud development in *Eya1* ^*−/−*^ FUP. Asterisks indicate stunted CVP and FOP epithelium. **(F)** Statistical analysis showing the height of crypts across the entire CVP from three entire CVPs. Scale bars: 1 mm **(A)**, 200 μm (lower magnification in B), 50 μm (higher magnification in B), 30 μm (FUP in C), 90 μm (CVP in C) and 50 μm (FOP in C).

During the course of our phenotypic analysis of craniofacial defects associated with *Eya1* deficiency, we noticed that the tongues of *Eya1* ^*−/−*^ mice that die at birth (Xu et al., 1999) were severely reduced in size compared to wild-type control littermates (Figure 1A). Although not directly related to the development of taste papillae, the tongue primordium forms from two lateral lingual swellings called the tongue buds, which originate from the floor of the first and second pharyngeal arches at E11.5 (Paulson et al., 1985; Shuler and Dalrymple, 2001; Torii et al., 2016). In *Eya1* ^*−/−*^ mice, the overall size of the tongue was noticeably smaller as early as E12.5 and drastically reduced in size by E18.5 compared to wild-type littermates (Figure 1A). We examined a total of 6-8 embryos of each genotype, and the phenotype was reproducible in all embryos. Thus, *Eya1* is required for normal tongue formation during embryonic development.

The three types of taste papillae—FUP, CVP, and FOP—also displayed abnormal morphology or growth arrest (Figures 1B, D). The wild-type tongue has well-developed FUP with surrounding flattened keratinocytes near the taste pore (arrow, Figure 1B). In *Eya1* ^*−/−*^ embryos, the FUP formed, but the cells did not appear tightly clustered (arrowhead, Figure 1C), suggesting defective taste bud differentiation. The epithelial trenches of the CVP (indicated by bars) failed to invaginate deeply into the mesenchyme and appeared shortened but were still able to give rise to von Ebner’s glands. However, fewer von Ebner’s gland-like structures were identifiable in serial sections of the entire papilla regions compared to those typically observed in wild-type controls (Figure 1D). The CVP epithelium also appeared stunted (asterisk, Figure 1D) compared to the control. Likewise, the FOP trench invagination and papillary epithelium development were also disrupted (Figure 1D). We measured the height of the taste crypt in serial sections across the entire papilla for three CVPs, and the data confirmed shortened taste crypt in *Eya1* ^*−/−*^ (Figure 1F). These data indicate that Eya1 activity is required for the development of epithelium-derived taste papillae.

### *Eya1*-deficient taste papillae lack nerve innervation

We then analyzed taste papillae at earlier stages. The development of taste papillae initiates around E12.5 with the emergence of taste primordia as epithelial thickenings or taste placodes (Supplementary Figure S1A–C). In *Eya1* ^*−/−*^ mice, taste placodes were formed at E12.5 (Supplementary Figure S1A–C), indicating that *Eya1* is not necessary for the initial formation of taste placodes.

Previous studies have demonstrated that gustatory nerves are essential for the development of taste papillae, with taste buds in FUP innervated by the VIIth nerve and those in CVP and FOP innervated by the IXth nerve (Gibbons and Sadiq, 2022). Gustatory nerve fibers are critical for taste bud cell renewal [reviewed in (54)], and ablation or poor growth of these fibers can result in abnormal and distorted papillae during late embryonic and early postnatal stages (Barlow et al., 1996; Oakley and Witt, 2004). A recent study has also highlighted the importance of innervation in the embryonic development of taste buds, as genetic ablation of Neurog2, a key transcription factor involved in the development of neurogenic placode-derived sensory neurons, disrupts taste bud formation (Fan et al., 2019). Given that our previous work demonstrated severe defects in neurogenic placode-derived cranial ganglia, including geniculate (VIIth) and petrosal (IXth) ganglia, in *Eya1* ^*−/−*^ mice (Zou et al., 2004), we examined whether gustatory nerve innervation was disrupted in *Eya1*-deficient mice. Immunostaining for the neuronal marker TUBB3 at E15.5 and E18.5 revealed that FUP taste buds on the top were fully innervated by nerve fibers in wild-type mice by E18.5 (arrow, Figure 1C; Supplementary Figure S1D), whereas only a few TUBB3^+^ nerve fibers were observed in the mutant FUP region at E15.5 and E18.5 (Supplementary Figure S1D; Figure 1C, arrowhead). The CVP and FOP regions of wild-type embryos at E15.5 and E18.5 showed nerve fiber association, as evidenced by TUBB3 staining (Supplementary Figure S1E, F; Figure 1E). In contrast, the mutant tongue had significantly fewer nerve fibers in the CVP and FOP regions (Supplementary Figure S1E, F; Figure 1E, asterisks indicate stunted papillary epithelium). Therefore, the lack of nerve contacts in the epithelial cells of taste papillae could disrupt taste bud formation and lead to a defective differentiation program of taste bud cells in the mutant.

### Expression of Six1 in the gustatory papilla epithelium depends on *Eya1*

**FIGURE 2:**
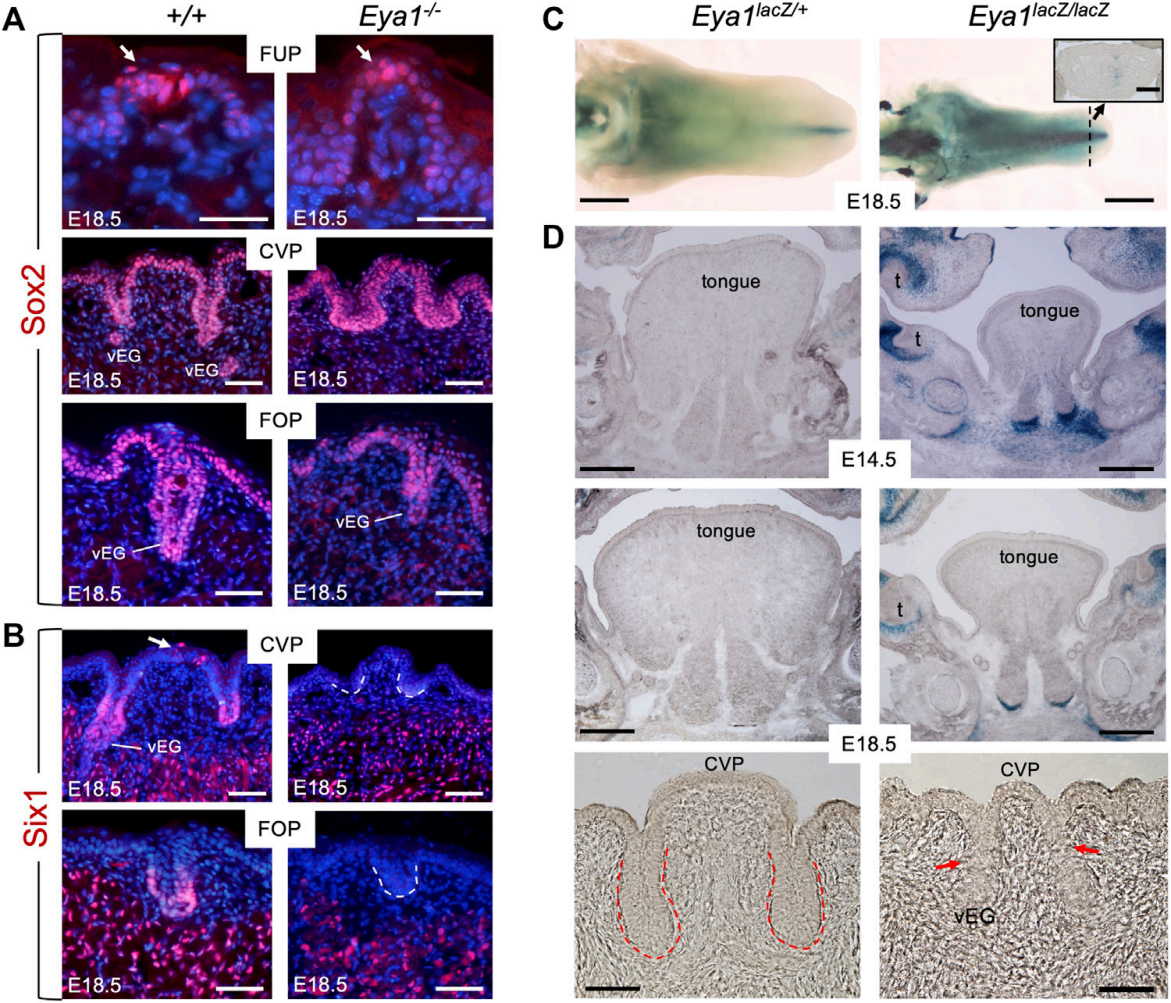
Loss of Six1 expression in CVP and FOP in *Eya1-*deficient tongues and absence of *Eya1* expression in embryonic tongues. **(A)** Immunostaining on frontal sections showing Sox2 expression in E18.5 wild-type and *Eya1* ^*−/−*^ FUP, CVP and FOP regions. Arrows indicate well-developed taste buds with clustered cells in wild-type FUP but not in *Eya1* ^*−/−*^. **(B)** Immunostaining on frontal sections showing Six1 expression in CVP epithelium (arrow) and trenches, FOP trenches, von Ebner’s gland and muscle cells in E18.5 wild-type, but its expression in the papilla and trench epithelium is lost in *Eya1* ^*−/−*^. **(C)** Whole-mount X-Gal staining of *Eya1* ^*LacZ/+*^ and *Eya1* ^*LacZ/LacZ*^ tongues at E18.5. **(D)** Frontal sections showing X-Gal staining of *Eya1* ^*LacZ/+*^ and *Eya1* ^*LacZ/LacZ*^ tongues at E14.5 and E18.5. Red arrows point to the end of the trench epithelium, which was followed by a cluster of epithelial cells. Abb.: t, tooth; vEG, von Ebner’s gland. Scale bars: 30 μm (for top panels of FUP in A), 50 μm (for CVP and FOP in A,B), 1 mm **(C)** and 300 μm **(D)**.

Next, to define the molecular basis underlying abnormal papilla morphogenesis, we performed marker gene analysis. We previously found that Eya1 acts upstream of Sox2 and Six1 in a genetic network controlling the formation of sensory organs in the inner ear (Ahmed et al., 2012a; Li et al., 2020). Therefore, we examined the regulatory relationship between these genes in the taste system. *Sox2* is expressed in the lingual epithelium and is required for the differentiation of epithelial progenitors into taste cells (Okubo et al., 2006). Immunostaining showed that Sox2 was strongly detected in well-developed taste bud cells but was relatively weak in surrounding epithelial cells in the FUP region of the wild-type tongue at E18.5 (Figure 2A). In *Eya1* ^−/−^ tongues, Sox2 expression was also strongly detected in the FUP epithelial cells, but the taste bud did not appear as onion-shaped, with tightly clustered and elongated cells (Figure 2A), similar to that seen histologically (Figure 1C). Although morphologically abnormal, the levels of Sox2 expression in the trenches and epithelium of the CVP and FOP as well as in von Ebner’s gland appeared unaffected in *Eya1* ^*−/−*^ (Figure 2A). Thus, Sox2 expression in the taste epithelium is *Eya1*-independent.

*Six1* is expressed in the trenches of the CVP and FOP at E18.5, previously detected by ISH (Suzuki et al., 2010b). Consistent with this, immunostaining detected Six1 expression at the bottom of the trench epithelium and von Ebner’s glands at E18.5 (Figure 2B). We also observed Six1^+^ cells in the top CVP epithelium (arrow, Figure 2B). In contrast, *Eya1* ^*−/−*^ tongues lacked Six1 expression in these structures, but Six1 expression was detectable in the muscle cells (Figure 2B). This suggests that *Eya1* is required for *Six1* expression in the epithelium of trenches and papillae during development.

Since the expression of *Eya1* during embryonic tongue and taste organ development has not been studied, we performed X-Gal staining of tongues isolated from *Eya1* ^*LacZ*^ knockin reporter mice (Zou et al., 2008; Xu et al., 2014). To our surprise, whole-mount or section staining at E12.5-18.5 failed to detect β-Gal activity in the developing tongue epithelium or taste papillae of either *Eya1* ^*LacZ/+*^ heterozygotes or *Eya1* ^*LacZ/LacZ*^ homozygotes (Figures 2C, D; Supplementary Figure S2A). In contrast, some β-Gal activity was observed near the midline in the tip region (inset panel in Figure 2C) and other regions of the oral cavity (Figure 2D). Since *Eya1* is not actively expressed in the tongue epithelium and epithelium-derived taste papillae, it is unlikely that *Eya1* plays a direct role in the morphogenesis of the taste papillae during embryonic development. Therefore, the abnormal growth of taste papillae observed in *Eya1* ^*−/−*^ may be primarily due to the absence of associated afferent nerves.

### *Eya1*-expressing pharyngeal ectoderm/endoderm give rise to tongue epithelium and taste papillae

**FIGURE 3:**
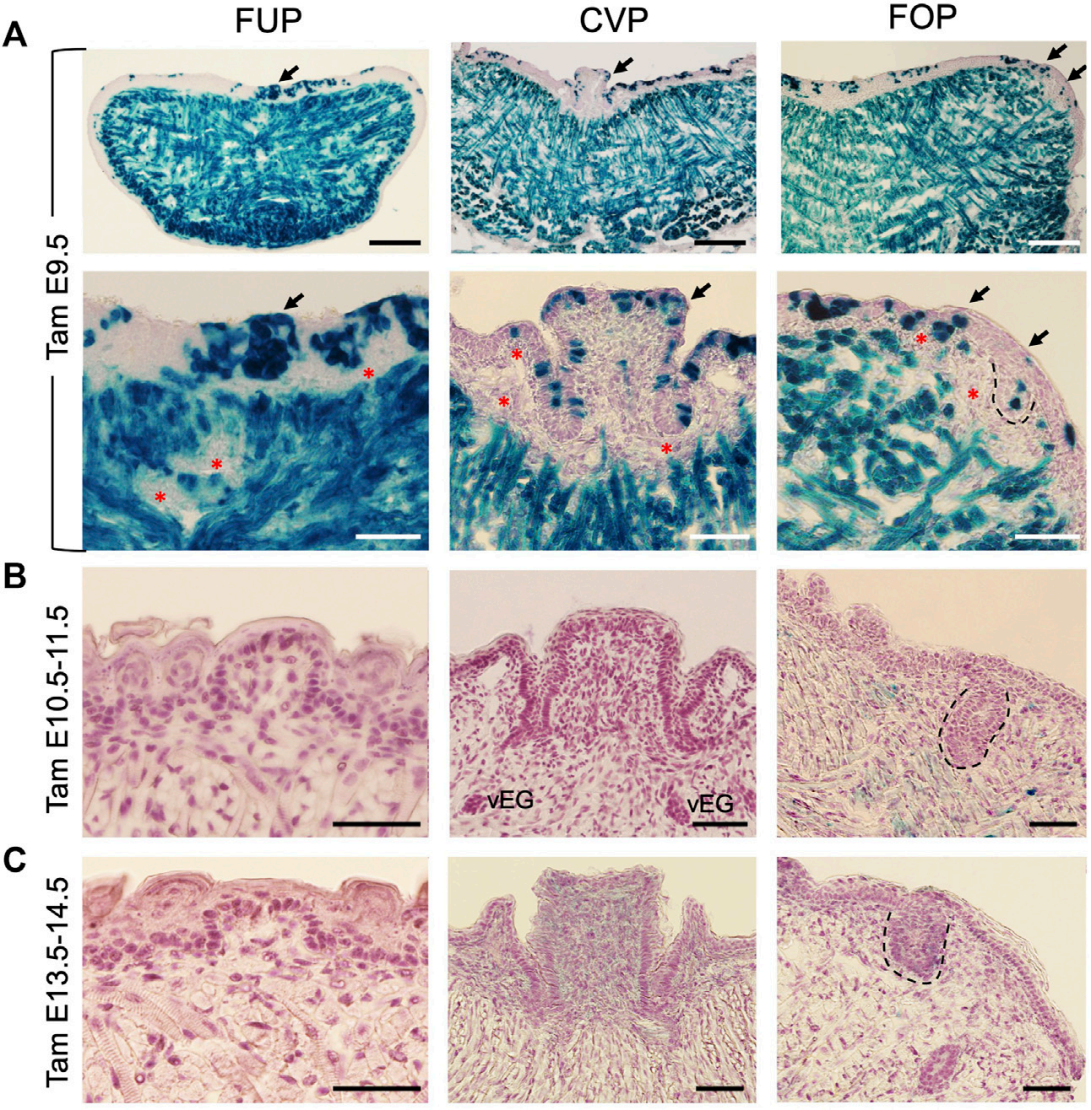
Lineage tracing reveals the contribution of *Eya1-*expressing progenitors before E10.5 to tongue epithelium, taste papillae, and tongue musculature. **(A–C)** X-Gal stained frontal sections of *Eya1* ^*CreER*^ *;R26R* ^*LacZ*^ at E17.5–18.5 showing labeled cells in the tongue muscles, lingual epithelium, and taste papilla regions when tamoxifen (tam) was administered at E9.5 **(A)**, but not at E10.5 and 11.5 **(B)** or E13.5 and 14.5 **(C)**. Bottom panels in A show a higher magnification of areas indicated by arrows. The FOP was outlined by dashed lines. Red asterisks indicate unmarked mesenchymal cells. Abb.: vEG, von Ebner’s gland. Scale bars: 200 μm for the top panels in A and 50 μm for all other panels.

Previous lineage tracing studies have indicated that taste papillae located in the anterior and posterior tongue have different embryonic origins, with FUP being ectoderm-derived and both CVP and FOP being endoderm-derived (Engert et al., 2009; Rothova et al., 2012). We showed that *Eya1* is coexpressed with Six1 in pharyngeal endoderm, ectoderm and mesenchyme at E9.5-10.5 (Xu et al., 1997b; Xu et al., 2002), but the expression of *Six1* in the pharyngeal endoderm and ectoderm is absent in *Eya1* ^*−/−*^ embryo (Xu et al., 2002; Zou et al., 2006b). This led us to hypothesize that early *Eya1*-expressing pharyngeal progenitors might contribute to tongue formation and that in the absence of *Eya1*, these progenitors might not be able to undergo normal cellular proliferation. To test this, we performed lineage tracing using *Eya1* ^*CreE*R^ (Xu et al., 2014) and Cre-dependent *R26R* ^*LacZ*^ reporter by administering tamoxifen at E9.5-E14.5 and staining for β-Gal activity in the tongue at E17.5-18.5. Indeed, one dose of tamoxifen administration at E9.5 induced *LacZ*-marked cells in the tongue epithelium and epithelium-derived taste papillae (Figure 3A). Additionally, since both genes are also coexpressed in somites, where Six1 has a primary role in myogenesis (Laclef et al., 2003), the tongue muscle cells, which predominantly derive from the myoblasts that originate in the occipital somites (Iwata et al., 2013), were marked by β-Gal (Figure 3A). However, no labeled cells were observed in some cells surrounding the muscles or the subepithelial mesenchyme adjacent to the epithelium (red asterisks, Figure 3A). These mesenchymal cells also do not appear to express Six1 (Figure 2B). Notably, however, tamoxifen administration from E10.5 to E14.5 failed to induce *Eya1*-lineage cells in the tongue and taste papillae, indicating that *Eya1*-expressing progenitors from E10.5–14.5 did not contribute to the formation of tongue and taste papillae (Figures 3B, C). These data also confirmed the absence of *Eya1* expression in the developing tongue and taste papillae (Figures 2C, D; Supplementary Figure S2A). Since the peak of CreER activation occurs over a subsequent period of 12–24 h (Danielian et al., 1998; Zervas et al., 2004), and the efficiency of CreER activation is affected by the timing and amount of tamoxifen received by different cells, the stage of cell development/differentiation and the age of the animals, multiple doses of tamoxifen treatment are commonly used in genetic ablation studies. Thus, one dose of tamoxifen treatment may not be sufficient to induce simultaneous CreER activation in all *Eya1*-expressing progenitors, explaining why not all epithelial cells in taste papillae and trenches were β-Gal^+^. Nonetheless, these results suggest that early *Eya1*-expressing progenitors at E9.5–10.5 can give rise to tongue epithelium, taste papillae and tongue muscle tissues.

**FIGURE 4:**
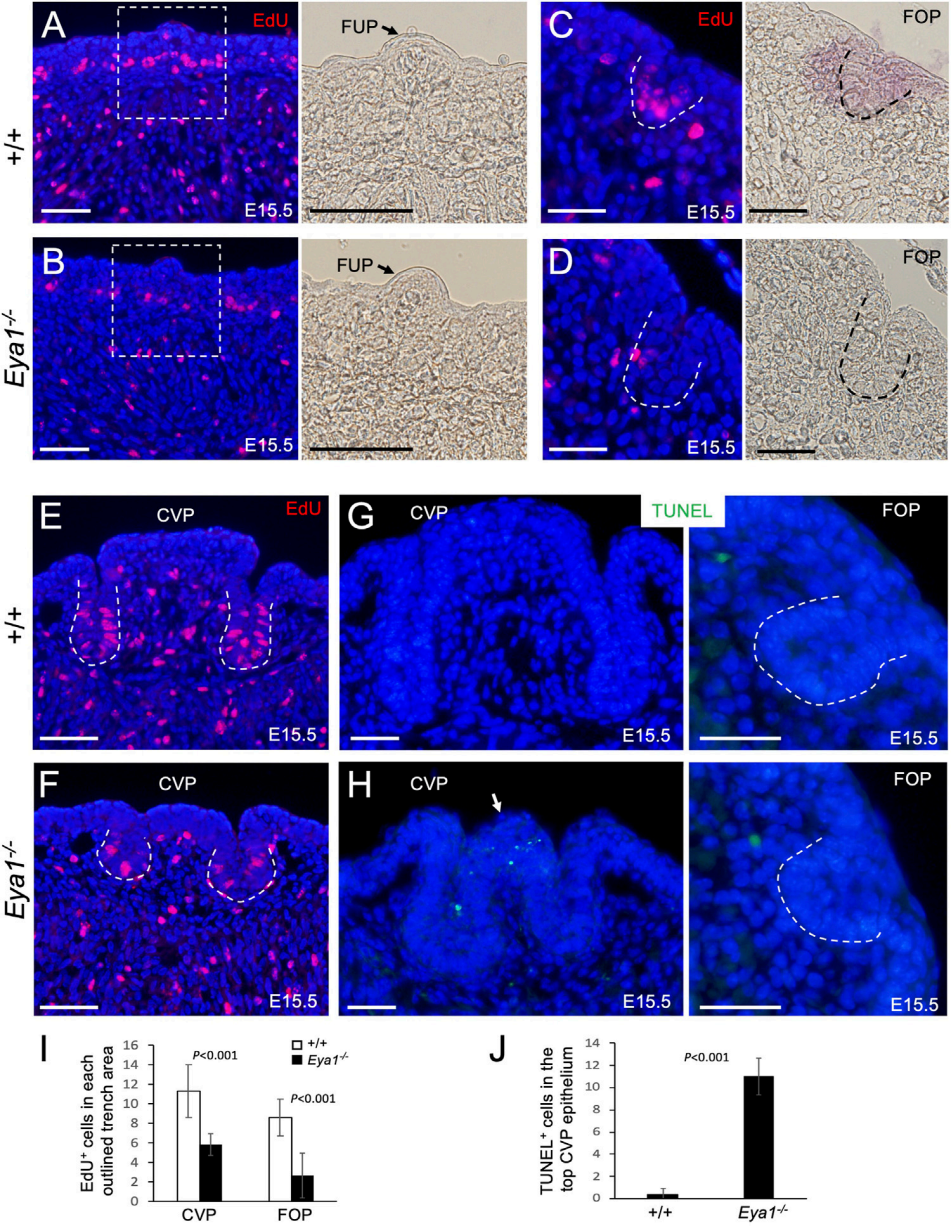
Reduced cell proliferation in the proliferating zone of the CVP and FOP trenches and increased apoptosis in the CVP epithelium of *Eya1* ^*−/−*^ tongues. **(A–F)** EdU (red) on sections of FUP, CVP and FOP regions from E15.5 tongues injected with EdU 1.5 h before harvesting. Right panels in A,B are bright-field images showing higher magnification of the boxed areas and the arrow in A,B points to FUP. Invaginating trenches of FOP **(C, D)** or CVP are outlined by dashed lines. Right panels in C,D are bright-field images. **(G, H)** TUNEL assay on sections of the CVP and FOP regions from E15.5 tongues showing increased apoptotic cells (green) in the CVP epithelium on the top (arrow). **(I)** Number of EdU^+^ cells in the outlined trenches (see Methods for quantification). *p*-values were measured for +/+ and *Eya1* ^*−/−*^ using two-tailed Student’s t-test. **(J)** Quantitative analysis of the number of TUNEL^+^ cells in the CVP epithelium region. Scale bars: 50 μm **(A, B, E, F)** and 30 μm **(C, D, G, H)**.

Next, we investigated whether the early progenitors had a defect in cell proliferation or survival associated with *Eya1* deficiency. To measure cell proliferation, we injected the mitotic tracer 5-ethynyl-2′-deoxyurindine (EdU) at E15.5 and harvested the tongue 1.5 h after injection. EdU-labeling assays confirmed that while EdU incorporation appeared to be reduced in the muscle cells, EdU-incorporated epithelial cells in the taste papilla regions, especially at the bottom of the growing CVP and FOP trenches, were significantly reduced in the mutant compared to the control littermates (Figures 4A–F). We counted EdU^+^ cells in the CVP and FOP trenches, and quantitative analysis indicated that EdU-incorporated cells in the mutant CVP and FOP trenches were reduced to ∼52.2% (CVP trench) or ∼40.7% (FOP trench) of those in the littermate controls (Figure 4I). Thus, in the absence of *Eya1*, the trench epithelial progenitor cells are likely unable to undergo normal proliferation and expansion, which may partially lead to the growth defect of the trench epithelium.

We also performed a TUNEL assay to investigate if the tongue progenitor cells undergo abnormal cell death and found that the number of apoptotic cells was only increased in the top epithelium of the mutant CVP at E15.5 (∼27.5-fold increase) compared to the littermate controls (Figures 4G, H, J). No abnormal apoptosis was observed in the growing trenches, other papilla regions, or the musculature. This suggests that CVP epithelial growth arrest may in turn lead to epithelial apoptosis.

### *Eya1* expression in postnatal CVP and FOP is important for taste cell differentiation

**FIGURE 5:**
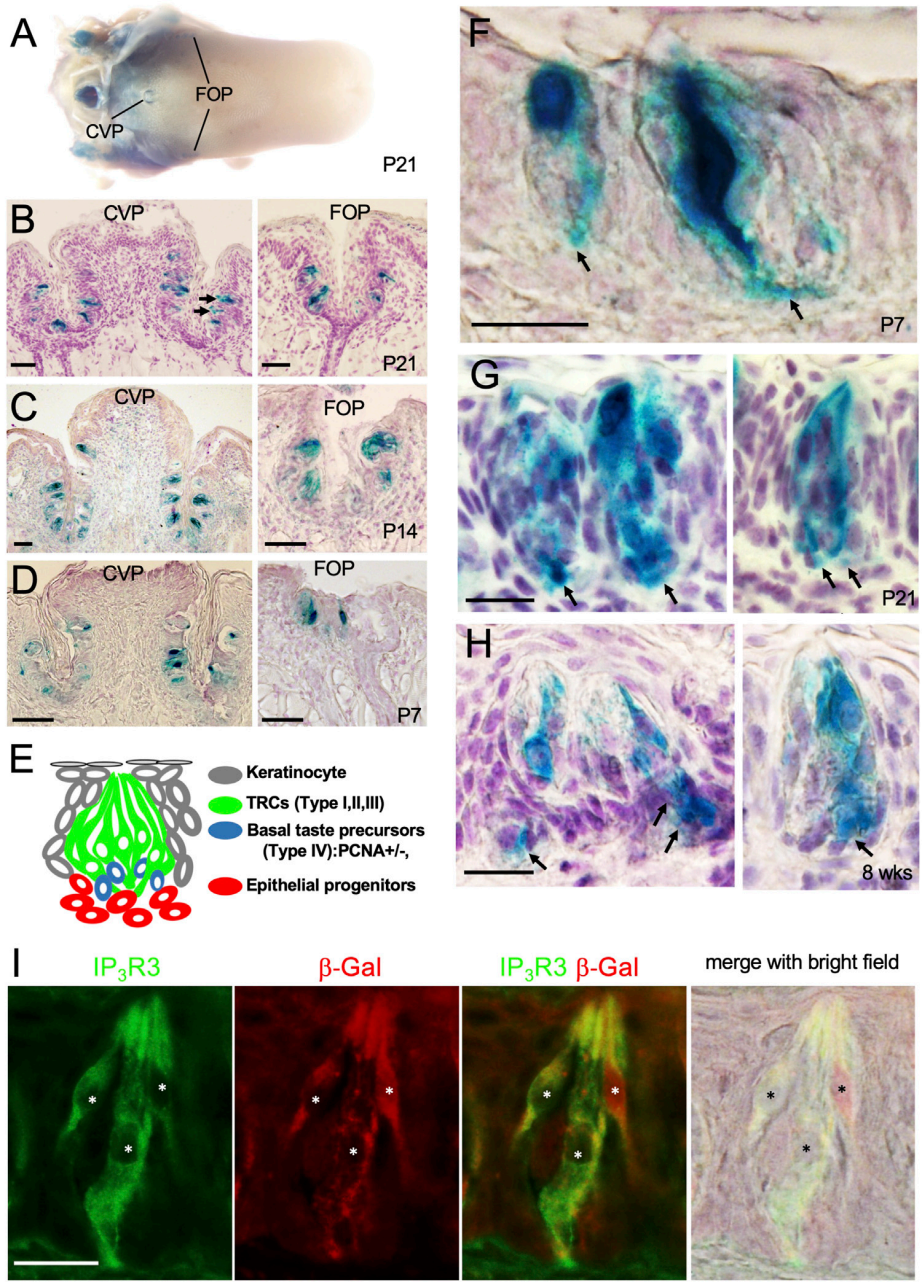
*Eya1* is expressed in a subset of taste cells and some basal progenitors after birth. **(A)** Whole-mount X-Gal staining of the tongue in *Eya1* ^*lacZ/+*^ mice at P21. **(B)** Sections of X-Gal stained tongue in **(A)** showing the CVP and FOP regions. **(C, D)** X-gal stained sections of CVP and FOP regions at P14 **(C)** and P7 **(D)**. **(E)** Schematic drawing showing different types of taste bud cells. **(F–H)** X-Gal stained sections showing β-Gal^+^ cells in the CVP taste buds at P7 **(F)**, P21 **(G)**, and 8 weeks **(H)**. Arrows point to basal progenitors. **(I)** Co-immunostaining for β-Gal and IP_3_R3 in taste buds at 8 weeks. Asterisks indicate IP_3_R3^+^ cells are also β-Gal^+^. Scale bars: 50 μm **(A–D)** and 20 μm **(F–I)**.

As noted earlier, *Eya1* has been reported to be expressed in a subset of Pou2f3^+^ Type II TRCs and some immature precursors in the endoderm-derived CVP and ectoderm-derived FUP but not in the endoderm-derived FOP at 8–12 weeks of age (Ohmoto et al., 2021). To test whether Eya1 regulates taste cell differentiation, we first clarified the expression of Eya1 at various postnatal stages. X-Gal staining of *Eya1* ^*LacZ/+*^ tongues at 3 weeks to adulthood showed weak β-Gal activity in taste buds of CVP and FOP (Figure 5A), but no β-Gal activity was observed in the ectoderm-derived FUP at any stage after birth (Figure 5A). X-Gal staining on sections confirmed β-Gal activity in the endoderm-derived CVP and FOP taste buds (Figure 5B). Analysis of P14 and P7 tongues also revealed β-Gal activity in the CVP and FOP taste buds (Figures 5C, D). Thus, our analysis redefines the expression of Eya1 as restricted to the endoderm-derived CVP and FOP taste buds located at the back of the tongue, but not the ectoderm-derived FUP situated in the anterior part of the tongue.

In the adult tongue, taste buds are maintained by continuous renewal of cells from surrounding epithelial stem progenitors throughout life. The taste bud cells are classified into four types: elongated and tightly clustered Type I, II, and III TRCs, and IV basal postmitotic precursors (Figure 5E) (Feng et al., 2014; Barlow and Klein, 2015; Yang et al., 2020; Finger and Barlow, 2021). Upon higher magnification of X-Gal-stained sections, strong β-Gal activity was observed in some taste cells and cells at the basal regions at P7, P21 and 8 weeks (arrows, Figures 5F–H). To clarify whether Eya1^+^ taste cells are a subset of Type II cells, we performed co-immunostaining with anti-β-Gal and -IP_3_R3 (a marker for Type II cells) and found that approximately 96% of β-Gal + cells were also IP_3_R3^+^ (Figure 5I; Table 1). This confirms that *Eya1* expression is predominantly restricted to Type II TRCs.

**TABLE 1:** Number of β-gal-positive cells and IP_3_R3-positive Type II cells in adult bud.

Total β-gal^+^/bud per 6 µm	Total β-gal^+^ IP_3_R3^+^/bud per 6 µm	Total IP_3_R3^+^/bud per 6 µm	% double positive cells per β-gal^+^ cells	% double positive cells per IP_3_R3^+^ cells
2.08 ± 1.17^a^		3.74 ± 1.35^b^		
β-gal^+^	β-gal^+^ IP_3_R3^+^	IP_3_R3^+^		
3	73	53	96.1	57.9

^a,b^The number represents an average of β-gal- or IP_3_R3-positive cells counted from 33–35 taste buds from 6 µm sections coimmunostained for β-gal/IP_3_R3.

Among the 76 β-gal-positive cells, 73 were also IP_3_R3-positive but 3 were IP_3_R3-negative.

Among the 126 IP_3_R3-positive cells, 73 were also β-gal-positive but 53 were β-gal-negative.

**FIGURE 6:**
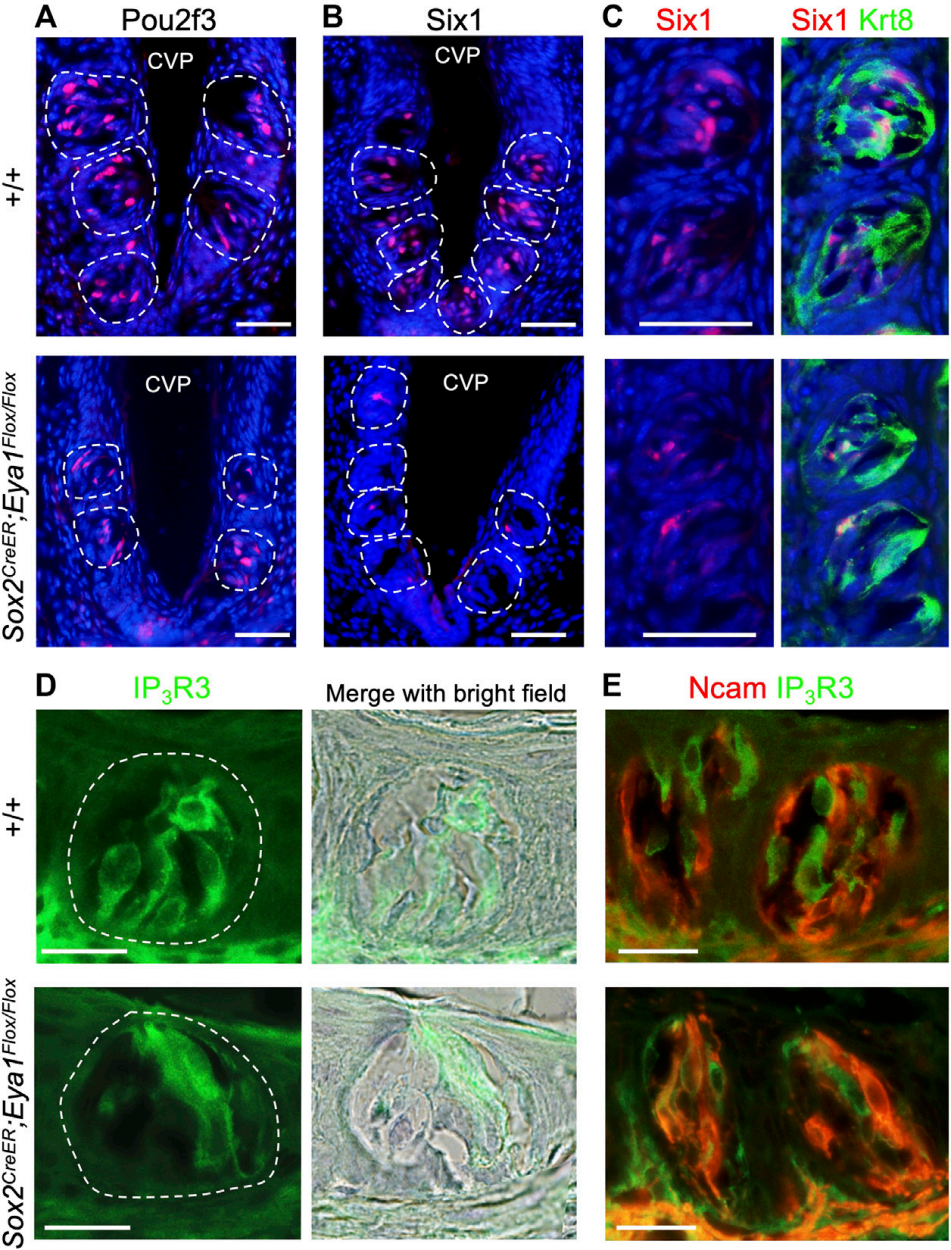
Conditional deletion of *Eya1* leads to a reduction in the numbers of Pou2f3^+^, Six1^+^ and IP_3_R3^+^ taste cells. **(A–E)** Immunostaining for Pou2f3 **(A)**, Six1 **(B)**, or co-immunostaining for Six1/Krt8 **(C)**, IP_3_R3 **(D)** or co-immunostaining for IP_3_R3/Ncam **(E)** in wild-type control and *Eya1*cKO taste buds at ∼6–8 weeks of age. Sections were also counterstained with diluted hematoxylin, and bright-field images were taken to outline the taste buds. Scale bars: 50 μm **(A–C)** and 20 μm **(D, E)**.

After confirming the expression pattern of *Eya1* in postnatal taste buds, we investigated whether it plays a role in the differentiation or maintenance of Type II TRCs by generating an *Eya1* conditional knockout (*Eya1*cKO). Previous studies have shown that Sox2 is expressed in some proliferative progenitors surrounding adult taste buds, postmitotic precursors, and taste cells (fewer Type II and Type III and more Type I) (Suzuki, 2008). We analyzed Sox2 expression in postnatal and adult taste buds in *Sox2* ^*GFP/+*^ knockin reporter mice (Arnold et al., 2011) and found that it is expressed in some basal progenitors and some taste cells (Supplementary Figure S3). As Eya1 was found to be expressed in many basal progenitor cells at P7-P21, we therefore used *Sox2* ^*CreER*^ and administered tamoxifen treatment at P15-P19. We harvested tongues at ∼6 weeks and immunostained for markers labeling different types of TRCs. Pou2f3 is specifically expressed in Type II TRCs, and inactivation of *Pou2f3* in mice results in loss of Type II TRCs and expansion of Type III cells (Matsumoto et al., 2011). In *Eya1*cKO, immunostaining showed a clear reduction in the number of Pou2f3^+^ cells in the taste buds (Figure 6A; Table 2), and the trench also appeared abnormal, with wider cavity space compared to the control (Figure 6A). Six1 has also been reported to be expressed in Type II cells (Suzuki et al., 2010a). Immunostaining for Six1 or co-staining with the pan-taste cell marker Krt8 (Mbiene and Roberts, 2003; Boggs et al., 2016) also showed a reduction in the number of Six1^+^ cells in the mutant taste buds (Figures 6B, C; Table 2). Immunostaining for IP_3_R3 (Golden et al., 2021) or co-immunostaining for IP_3_R3 and Ncam (a marker for Type III cells) further confirmed the reduction of Type II cells in *Eya1*cKO (Figures 6D, E; Table 2). However, no significant difference was observed in the number of Ncam^+^ Type III cells between wild-type and *Eya1*cKO mice (Figure 6E; Table 2). Thus, these results suggest that deletion of Eya1 activity in *Sox2*-expressing cells may affect gene expression levels or Type II cell turnover rates.

**TABLE 2:** Number of Type II cells per bud per 6 μm

	Pou2f3^+^ cells	% of Pou2f3^+^ cells in mutant vs. WT	Six1^+^ cells	% of Six1^+^ cells in mutant vs. WT	IP_3_R3^+^ cells	% of IP_3_R3^+^ cells in mutant vs. WT	Ncam^+^ cells	% of Ncam^+^ cells in mutant vs. WT
+/+	4.2 ± 1.7		3.3 ± 0.9		6.07 ± 1.1		2.47 ± 1.0	
*Eya1cKO*	2.1 ± 1.6^a^	50.0	0.8 ± 0.7^c^	24.2	2.93 ± 0.9^e^	48.3	2.73 ± 0.9^g^	110.5
*Eya2* ^*LacZ/LacZ*^	3.0 ± 0.8^b^	71.4	1.3 ± 0.9^d^	39.3	4.73 ± 1.0^f^	77.9	2.53 ± 1.1^h^	102.3

The numbers of Pou2f3^+^, Six1^+^ or 1P_3_R3^+^ Type II cells were counted per taste bud on 6 μm sections. Each value represents the mean ± SD. The percentage of Type II cells in each mutant to wild-type per taste bud per 6 μm was calculated. ^a-k^ *p*- value: 0.00023249^a^, 0.007140024^b^, 7.05702E-12^c^, 4.04295E-08^d^, 3.48768E-09^e^, 0.001932706^f^, 0.403282673^g^, 0.860078781^h^.

### *Eya2* is expressed in CVP and FOP during embryonic tongue development

**FIGURE 7:**
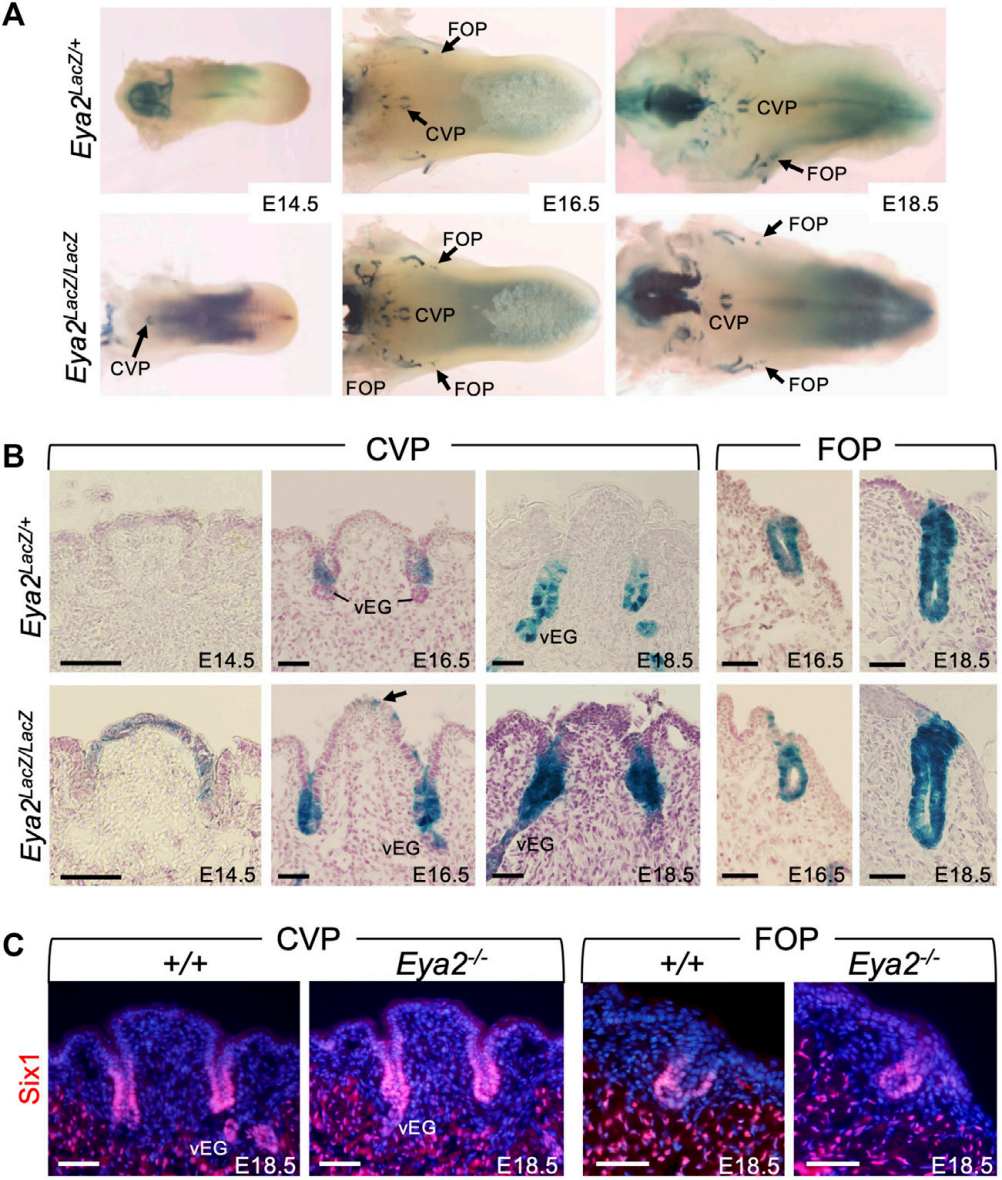
*Eya2* is expressed in the epithelium of CVP and FOP from the onset of papilla morphogenesis. **(A)** Whole-mount X-Gal staining of tongues from *Eya2* ^*LacZ/+*^ and *Eya2* ^*LacZ/LaZ*^ at E14.5, E16.5, and E18.5. **(B)** Frontal sections of whole-mount X-Gal stained tongues showing β-Gal activity the CVP epithelium and trenches, FOP trenches, and von Ebner’s gland in *Eya2* ^*LacZ/+*^ and *Eya2* ^*LacZ/LacZ*^ respectively. **(C)** Immunostaining on frontal sections showing Six1 expression in the trenches of CVP and FOP of wild-type and *Eya*2-null tongues at E18.5. Scale bars: 50 μm.

*Eya2* is broadly expressed in cranial sensory organs in an overlapping or complementary pattern with Eya1 during mouse development (Xu et al., 1997b; Zhang et al., 2021). *Eya2-*deficent mice are viable but develop hearing loss (Zhang et al., 2021), However, it has not been reported whether *Eya2* is expressed in the tongue and whether it plays a role in taste organ development. To address these questions, we first examined the expression pattern of *Eya2* during tongue development by performing X-Gal staining of *Eya2* ^*LacZ/+*^ or *Eya2* ^*LacZ/LacZ*^ from E14.5 to E18.5. Although we did not observe a clear difference in tongue appearance between *Eya2* ^*LacZ/+*^ and *Eya2* ^*LacZ/LacZ*^, we detected β-Gal activity in the CVP and FOP, but not in the FUP (Figure 7A; Supplementary Figure S2B). *Eya2* expression became detectable in the CVP epithelium at E14.5 as revealed by weak β-Gal activity in *Eya2* ^*LacZ/+*^ heterozygotes and stronger in *Eya2* ^*LacZ/LacZ*^ homozygotes (Figure 7B). As development proceeded, intense β-Gal activity was detected in the invaginating trenches and in the epithelium of CVP at E16.5-E18.5 (Figure 7B). At these stages, von Ebner’s glands appeared and showed strong *Eya2* expression (Figure 7B). Similarly, *Eya2* was expressed in the FOP trench epithelium (Figure 7B). Immunostaining for Six1 at E18.5 did not detect any significant differences in its expression levels between control and *Eya2*-deficient tongues (Figure 7C), suggesting that Six1 expression is not dependent on *Eya2*.

### *Eya2* is expressed in postnatal CVP and FOP

**FIGURE 8:**
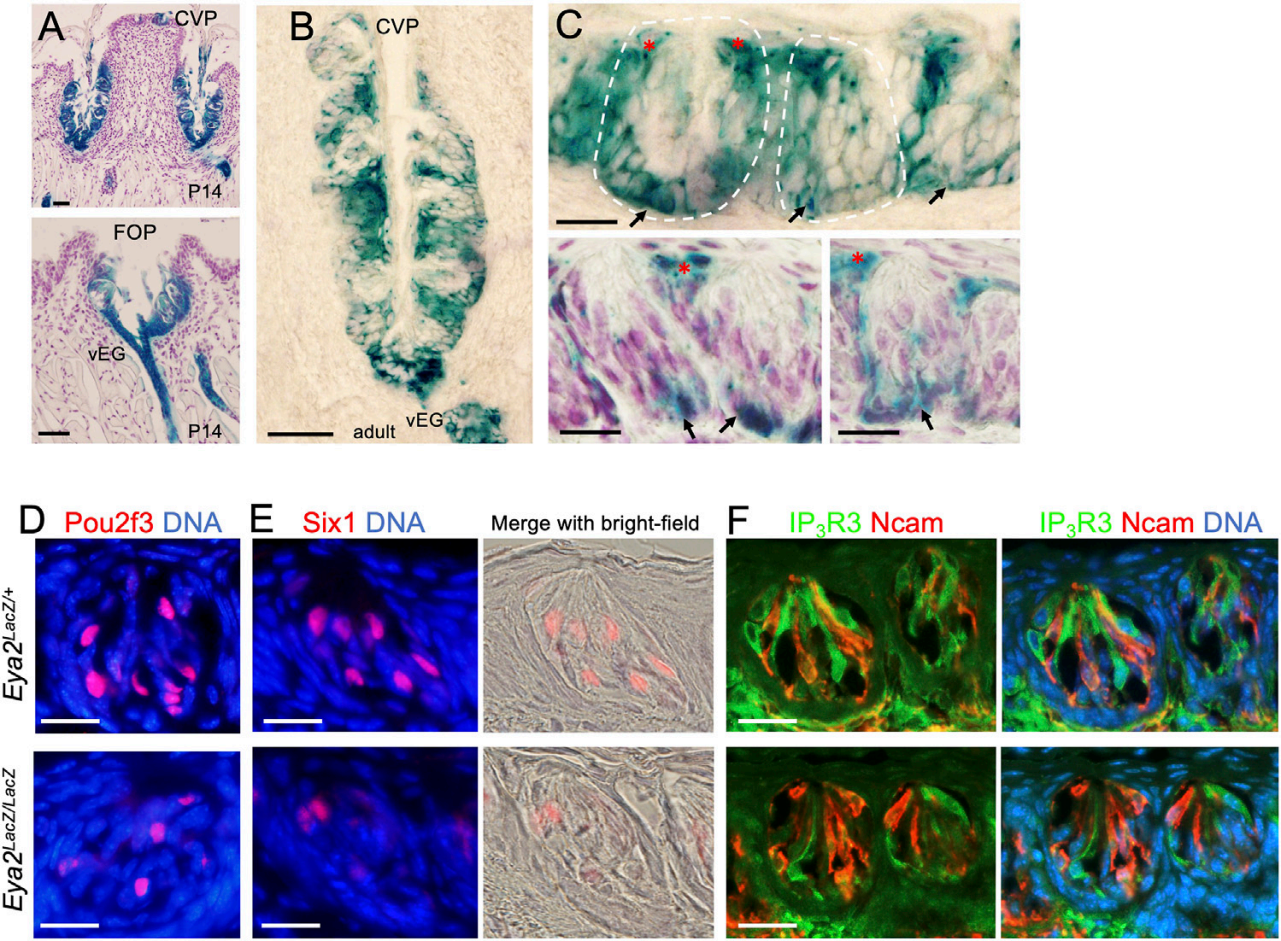
*Eya2* is expressed in the CVP and FOP taste buds, and *Eya2*-deficient mice show a reduced number of Type II taste cells. **(A, B)** X-Gal staining on sections showing broad expression of *Eya2* ^*LacZ*^ in the CVP and FOP trench epithelium at P14 **(A)** and the adult CVP trench epithelium **(B)**. **(C)** Higher magnification showing β-Gal^+^ cells in some basal progenitors (arrows) and cells surrounding the taste pore (red asterisks) and inside the taste buds. Some sections were counterstained with diluted hematoxylin (A and two lower panels in **(C)**. **(D–F)** Immunostaining for Pou2f3 **(D)**, Six1**(E)**, and co-immunostaining for IP_3_R3/Ncam **(F)** in wild-type control and *Eya2*-deficient adult taste buds. Scale bars: 50 μm **(A, B)** and 20 μm **(C–E)**.

After birth, *Eya2* continues to be widely expressed in the CVP and FOP but not in the FUP (Supplementary Figure S2C) and its expression spans the entire trench epithelium, including the taste buds and surrounding epithelium (Figure 8A). In the adult tongue, X-gal staining of sections from the *Eya2* ^*LacZ/+*^ CVP and FOP regions revealed β-Gal activity in some cells near the taste pores surrounding the buds (Figures 8B, C, red asterisks) and some cells in the basal regions (Figures 8B, C, arrows), but relatively weaker β-Gal activity was detected in a subset of taste cells (Figures 8B, C).

To investigate whether *Eya2* has a role in the differentiation of the TRCs in the adult CVP and FOP taste buds, we performed marker gene analysis. Similar to that observed in the *Eya1*cKO, the number of Pou2f3^+^, Six1^+^ or IP_3_R3^+^ Type II cells was also reduced in the mutant taste buds of the CVP and FOP (Figures 8D–F; Table 2). However, the number of Ncam^+^ Type III cells appeared unaffected in *Eya2*-deficient tongues (Table 2). Collectively, these results suggest that *Eya1* and *Eya2* may function together to regulate Type II TRC generation.

## Discussion

Transcription factors are known to spatiotemporally regulate gene expression programs, generating cellular diversity during development and maintaining cellular specificity over time. However, our understanding of how the taste system develops, and how taste cells are diversified and maintained, remains limited. The Eya transcription factor was initially identified as a critical regulator of compound eye formation in *Drosophila,* interacting with the homeodomain protein So/Six and Dachshund (Dach) to promote ectopic eye formation synergistically (Castillo-Azofeifa et al., 2018; Ohmoto et al., 2020). Genetically, *eya* is upstream of *so* because the expression of *so* is dependent on *eya* but not *vice versa* (Bonini et al., 1997; Pignoni et al., 1997; Halder et al., 1998). During mammalian development, members of the Eya gene family are coexpressed with the Six family genes in multiple organ systems, and the Eya-Six regulatory relationship is evolutionarily conserved (Wong et al., 2013; Xu, 2013). Although previous studies reported the expression patterns of Six1 and Six4 during embryonic taste papilla morphogenesis and Six1 expression in a subset of Type II TRCs in adult mice, it remains unexplored whether Six genes play any role in the differentiation of TRCs after birth or whether the Eya-Six regulatory network also operates in the taste system. In this study, we described previously unreported expression patterns of *Eya1* and *Eya2* in the taste system from embryonic stages to adulthood. By using *Eya1* ^*LacZ*^ or *Eya2* ^*LacZ*^ knockin reporter mice, we show for the first time that these two genes are predominantly expressed in the endoderm-derived CVP and FOP taste buds located at the back of the tongue. Furthermore, our analyses reveal an early role for *Eya1* in regulating embryonic tongue patterning and a later role for *Eya1* and *Eya2* in Type II cell generation or maintenance. Our results also indicate the importance of the Eya-Six network in regulating gustatory system morphogenesis.

### Possible roles of Eya1 and Eya2 in the taste system during mouse embryonic development

Our expression studies and lineage tracing provide evidence that Eya1 is not actively expressed in the tongue from E10.5-E18.5, whereas *Eya1*-expressing progenitors at E9.5 give rise to tongue muscles, lingual epithelium, and epithelium-derived taste papillae. Without Eya1, these progenitors may not expand properly, resulting in growth arrest of the tongue and taste papillae. Thus, a primary function for *Eya1* during embryonic tongue development may be to maintain the competence of these different progenitor cells to expand and generate tongue musculature and epithelial structures. This was supported by the observation that *Eya1* ^*−/−*^ tongues showed reduced cell proliferation and increased apoptosis in the *Eya1*-derived lineage cells. Since a single dose of tamoxifen treatment at E9.5 resulted in a mosaic pattern of β-Gal activity in the tongue epithelium and taste papillae/trench epithelium (Figure 4), it will be interesting to explore whether earlier administration of tamoxifen from E8.5, E9.0 or E9.25 will lead to more β-Gal-labeled cells in the epithelium of taste papillae and trenches.

In *Eya1* ^*−/−*^ tongues, we observed that Six1 expression was absent in the taste papillae and trench epithelium but remained present in the muscle cells (Figure 2). This finding is consistent with previous reports that *Six1* expression is lost in the pharyngeal endoderm and ectoderm but not in the somites of *Eya1* ^*−/−*^ embryos (Xu et al., 2002; Giordani et al., 2007; Grifone et al., 2007; Niro et al., 2010). Additionally, *Six1-*null mice exhibit stunned trench formation in the CVP and FOP and abnormal taste papilla morphogenesis (Suzuki et al., 2010b). Our results further suggest that Eya1 is crucial for cranial neurogenesis (Zou et al., 2004) and that *Eya1* ^*−/−*^ tongues lack contact with nerve endings in developing papillae and trenches (Figure 1; Supplemental Figure S1), indicating a role for *Eya1* in taste papilla development and differentiation. Interestingly, *Six1* expression is also *Eya1*-dependent in cranial sensory neurogenesis (Zou et al., 2004), and *Six1* ^*−/−*^ taste papillae show a decreased nerve supply (Suzuki et al., 2010b). However, unlike *Eya1*, *Six1* is actively expressed in taste papillae and trenches during their morphogenesis. Thus, it is unclear whether impaired trench formation and abnormal taste papilla morphogenesis in *Six1* ^*−/−*^ mice is due to the loss of early Six1 expression in pharyngeal progenitors before E10.5, *Six1* expression during tongue morphogenesis, or both. Future inducible deletion of *Six1* in the tongue may clarify this issue.

In contrast to *Eya1*, we found that *Eya2*, which is not expressed in pharyngeal endoderm or ectoderm at E9.5–10.5, is specifically expressed in the endoderm-derived CVP and FOP, shortly after the onset of their morphogenesis. It is also expressed in von Ebner’s glands. However, if *Eya2* has a direct role in the development of these papillae and acts upstream of *Six1*, one might expect abnormal CVP and FOP development and loss of *Six1* expression in *Eya2* ^*−/−*^ mice. Instead, *Eya2* ^*−/−*^ mice did not show noticeable anomalies in the initial papillary elevation and trench invagination as well as the pattern of *Six1* expression. Thus, it is possible that the other two Eya family members, *Eya3* or *Eya4*, may be coexpressed with *Eya2* and function redundantly in tongue epithelial cells to regulate *Six1* expression during CVP and FOP morphogenesis at embryonic stages. Future studies are required to address this issue.

### Roles of Eya1 and Eya2 in taste type II cells

Our analysis has shown that *Eya1* is not expressed in the ectoderm-derived FUP distributed in the anterior part of the tongue. Instead, its expression is restricted to the endoderm-derived CVP and FOP taste buds in the posterior tongue, which differentiate postnatally. As *Eya2* expression is also restricted to these endoderm-derived CVP and FOP taste buds, these two genes may function synergistically to regulate the CVP and FOP taste bud formation.

In the adult tongue, each taste bud is composed of three types of distinct TRCs. To maintain the taste bud tissues, the TRCs are all replaced over a period of weeks (∼every 2 weeks) by proliferative multipotent keratinocytes in the surrounding epithelium (Finger and Barlow, 2021). These epithelial stem/progenitor cells express the transcription factor Sox2 (Castillo-Azofeifa et al., 2018). The Sox2^+^ progenitors are induced to become postmitotic taste lineage-committed Type IV precursors located in the basal compartment of taste buds, and these committed precursors are capable of giving rise to Type I, II or III in adult buds (Miura et al., 2014). Type I cells make up approximately 50% of the differentiated cells in each bud and are glial-like cells thought to function as support cells within taste buds (Bartel et al., 2006; Yang et al., 2020). Type II cells make up approximately 20%–40% of TRCs detecting sweet, bitter, or umami stimuli, while Type III cells are the least common type sensitive to sour stimuli (Finger and Barlow, 2021). However, little is known about how distinct types of TRCs acquire their fate. To date, functional roles for transcription factors in TRC generation have only been demonstrated for Mash1 (Ascl1), which is required for Type III TRC development (Seta et al., 2011), and Pou2f3, which is required for Type II TRC generation (Matsumoto et al., 2011). *Pou2f3*-null tongues show loss of Type II TRCs and an increase in the number of Type III cells, with no detectable changes in the number of Type I cells. Six1 was previously reported to be expressed in a subset of Type II TRCs (Suzuki et al., 2010a); however, it remains unknown whether Six1 plays any role in the generation of Type II TRCs.

In this study, we found that *Eya1* is expressed in a subset of Type II cells, and conditional deletion of *Eya1* using *Sox2* ^*CreER*^ resulted in ∼50% reduction in the number of Pou2f3^+^ and IP_3_R3^+^ Type II cells in each taste bud (Table 2), whereas Six1^+^ Type II cells were reduced to ∼24% of those observed in the control. In contrast, although *Eya2* showed continuous expression in the CVP and FOP after birth, its expression level was higher in cells located in the basal regions and some cells near the taste pore but relatively weak in the taste cells. While less severe than that observed in *Eya1*cKO mice, since *Eya2*-deficient mice also showed reduced numbers of Type II TRCs (Table 2), *Eya2* may also have a role in the Type II TRC subtype lineage commitment. It should be noted that we used *Eya2* ^*LacZ/+*^ heterozygous samples for the expression analyses. Thus, future work using an anti-Eya2 specific antibody for immunohistochemistry, when it becomes available, will clarify whether Eya2 is also specifically expressed in Type II TRC subsets. Nonetheless, since deletion of either gene reduces the number of Type II cells, it is possible that these two genes may cooperate to regulate Type II TRC lineage commitment or maintenance. Therefore, they may function together to regulate the expression of Six1, which could explain why some Six1^+^ TRCs are still present in adult *Eya1*cKO or *Eya2-*deficient buds. Our data show that some Eya1-or Eya2-β-Gal^+^ cells are located in the basal region of taste buds, which leads us to speculate that these cells may be progenitors or precursors. Hence, it is plausible that these genes may be required to regulate gene expression programs that direct Type II TRC-specific lineage commitment. However, future studies using progenitor- or precursor-specific markers are necessary to confirm whether the basal β-Gal^+^ cells are proliferative progenitors or postmitotic taste precursors.

It will be interesting to investigate whether *Eya1* and *Eya2* have redundant roles in the CVP and FOP taste buds in *Eya1/Eya2* double mutant mice. Analysis of different types or subtypes of TRC marker genes in *Eya1* and *Eya2* double mutants will determine whether these two genes are involved in TRC differentiation. Future analyses on the possible role of other Eya genes and the Eya-Six pathway in regulating Type II TRC differentiation, as well as the regulatory relationship among Pou2f3, Eya, and Six genes, will help to elucidate the transcriptional network that controls the acquisition of Type II cell fate.

## Data Availability

The original contributions presented in the study are included in the article/Supplementary Material, further inquiries can be directed to the corresponding author.
