# Supplementary figures and images for: The role of Eya1 and Eya2 in the taste system of mice from embryonic stage to adulthood
Source: Front Cell Dev Biol. 2023 Apr 25;11:1126968. doi: 10.3389/fcell.2023.1126968 (PMC10167055; doi:10.3389/fcell.2023.1126968)

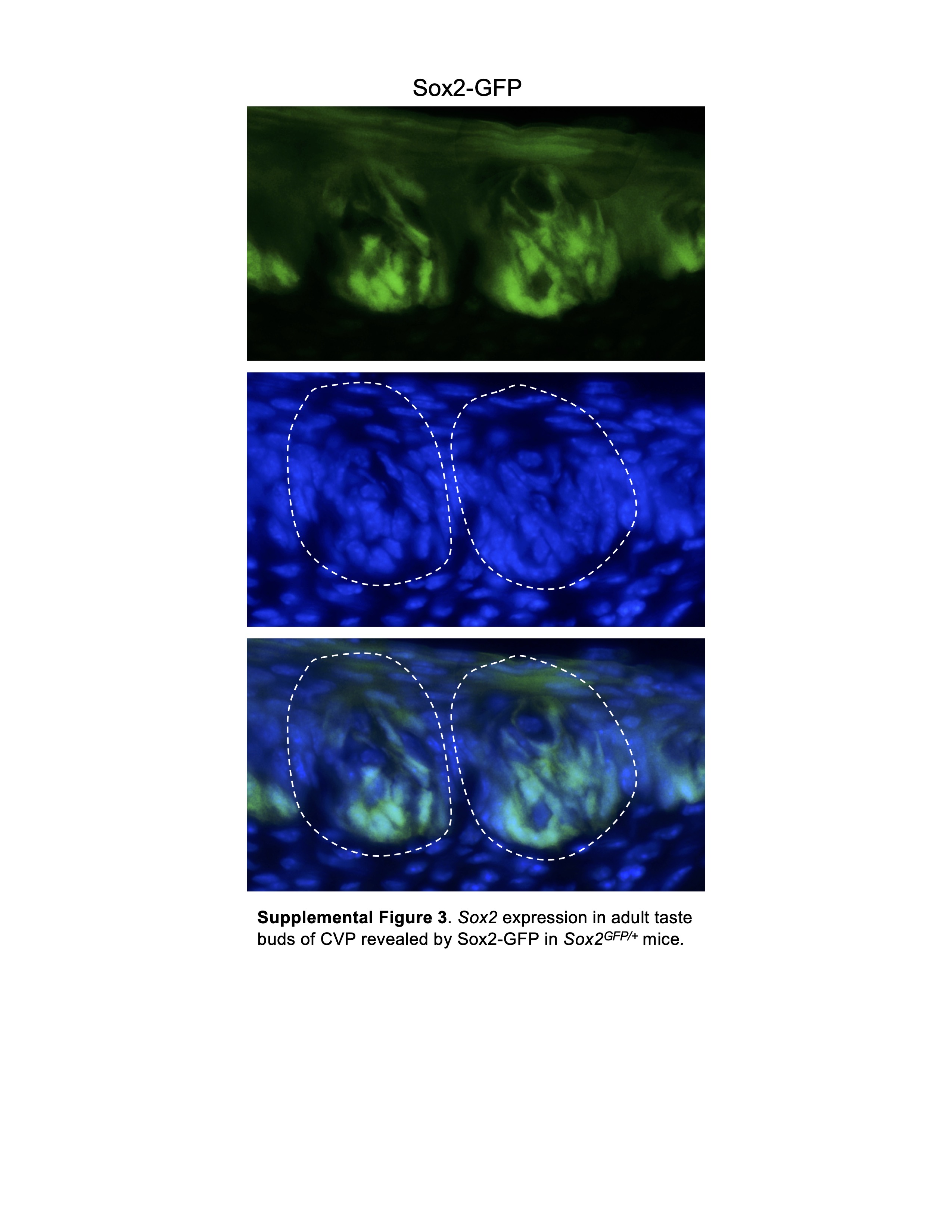

Supplement: Supplementary file 1 [file Image3.JPEG]

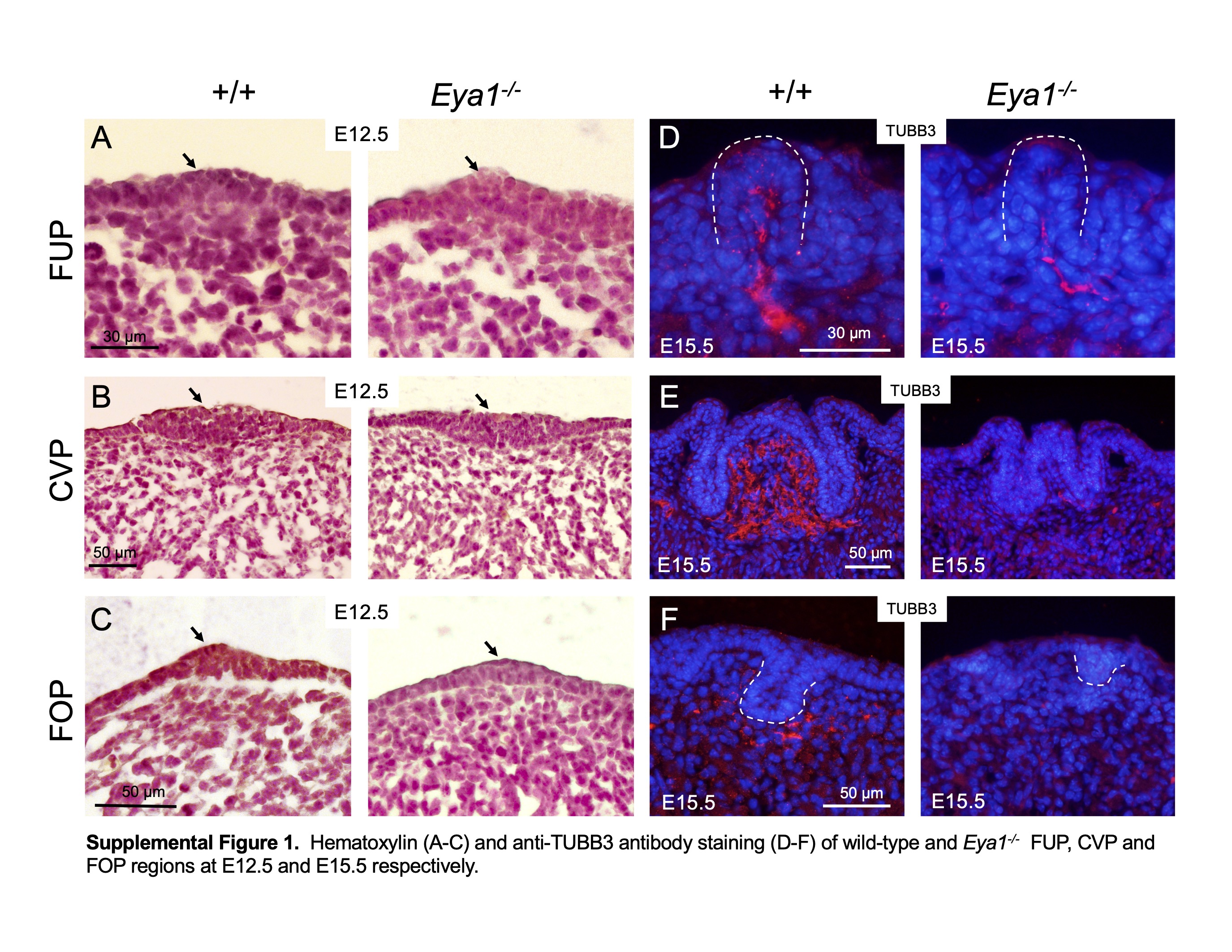

Supplement: Supplementary file 2 [file Image1.JPEG]

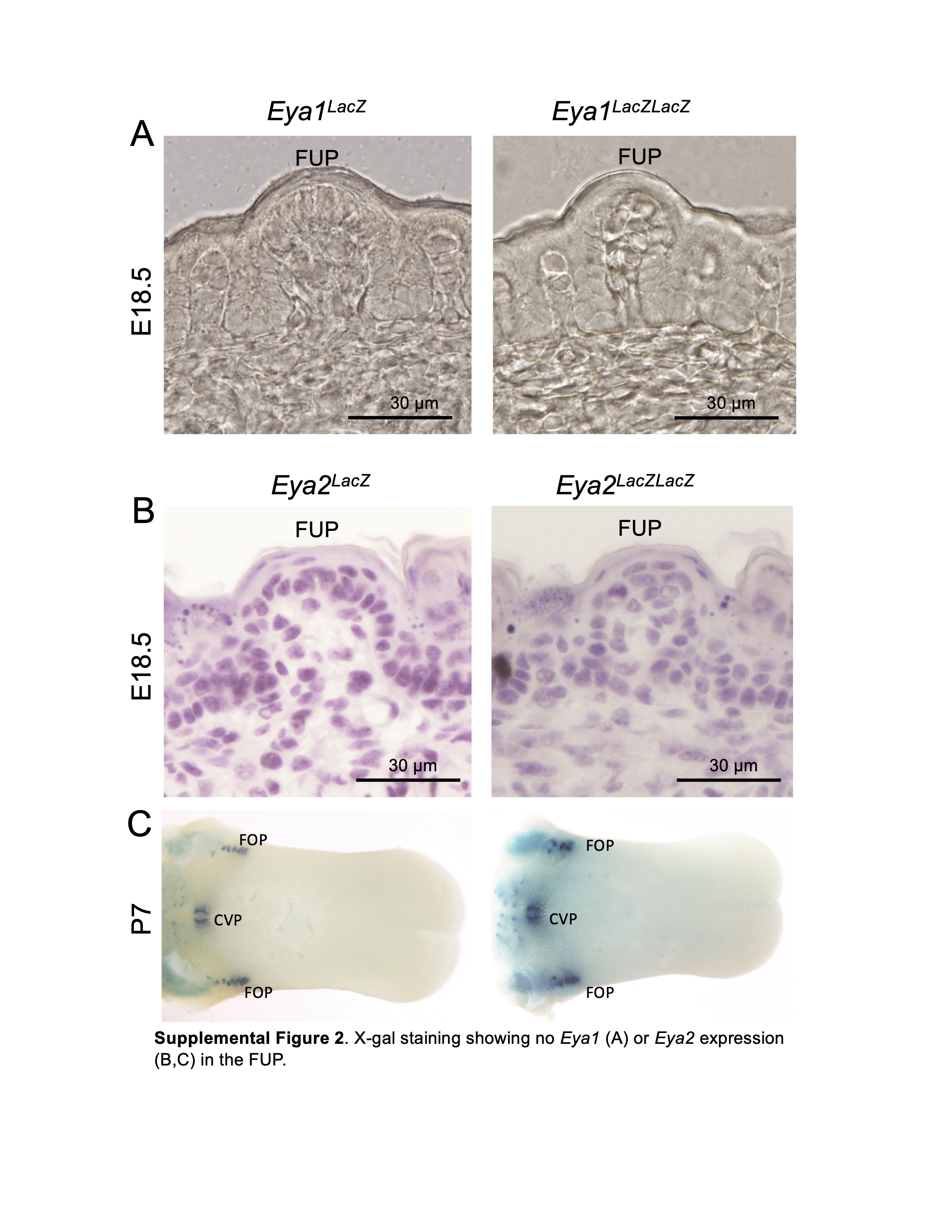

Supplement: Supplementary file 3 [file Image2.JPEG]
